# Synthetic switches of OGG1 control initiation of base excision repair and offer new treatment strategies

**DOI:** 10.1002/ctm2.1035

**Published:** 2022-08-29

**Authors:** Carlos Benitéz‐Buelga, Thomas Helleday, Maurice Michel

**Affiliations:** ^1^ Instituto de Investigaciones Biomédicas Alberto Sols (CSIC/UAM) Madrid Spain; ^2^ Sheffield Cancer Centre, Department of Oncology and Metabolism University of Sheffield Sheffield UK; ^3^ Science for Life Laboratory, Department of Oncology‐Pathology Karolinska Institute Stockholm Sweden

Although widely used in industry, organocatalysis has classically been limited to ex vivo application. In addition, the small molecule activation of enzymes has so far been exerted by allosteric control. Recently, we reported that small molecules can act as organocatalysts for the DNA repair enzyme 8‐oxoguanine DNA glycosylase 1 (OGG1). The underlying principle allows for a full control of enzymatic function with potential for alleviating oxidative stress to the genome or as a new strategy in cancer therapy.

## 1 ORGANOCATALYSIS AND SMALL MOLECULE ACTIVATION – A POWERFUL COMBINATION

Organic molecules that partake in a chemical reaction, increase its rate and exit it chemically unaltered are called organocatalysts.^1^ As a second class of reaction rate enhancing organic compounds, small molecule activators commonly operate as stabilizers of a protein conformation close to the transition state of an enzymatic reaction.^2^ A union of the two concepts has classically been considered unattractive, as partaking in the reaction would require binding to the enzymatic active site. This in turn would render the molecule an inhibitor as desired high compound concentration for high reaction turnover would compete with the originally intended substrate. However, this interpretation ignores enzymes with complex biochemistry, where substrate hydrolysis is achieved by consecutive steps of replacement and cleavage. Here, the inhibition or enhancement of single steps is conceivable.

**FIGURE 1:**
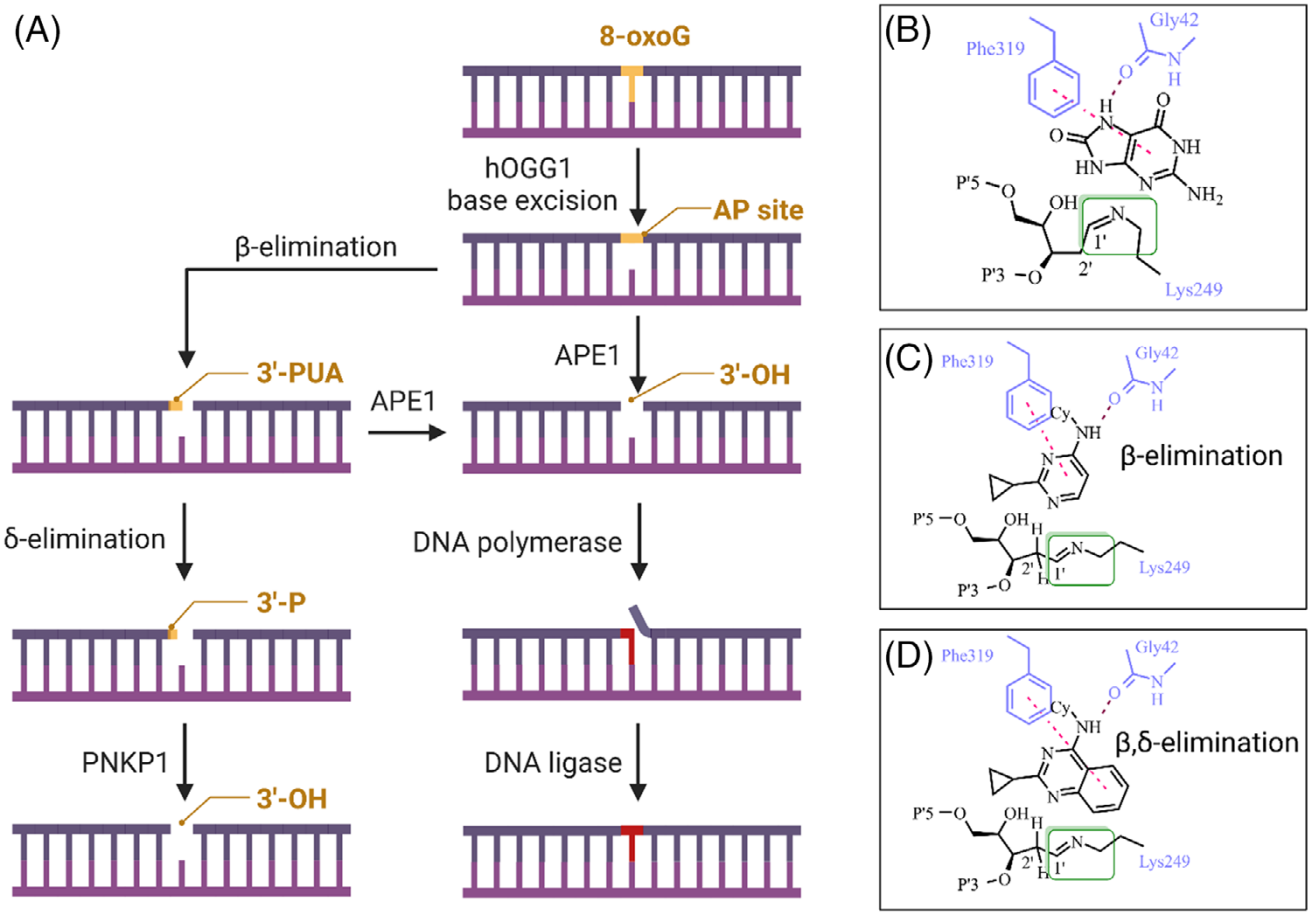
Mechanism of organocatalysis in base excision repair initiation by 8‐oxoguanine DNA glycosylase 1 (OGG1): (A) Upon base excision of 8‐oxoguanine (8‐oxoG) an abasic site (AP site) is generated, which in turn is a substrate for APE1. Even if OGG1 exerts its weak β‐elimination function, the generated product 3′‐phospho unsaturated aldehyde (3′‐PUA) will remain a substrate for APE1. In that way OGG1 function always depends on APE1. (B) After base excision, Lys249 and the opened form of the AP site form a Schiff base, also referred to as an imino group (green box). The cleavage of this group is the rate determining step in base excision repair initiation mediated by OGG1. Further, the formation of this functionality temporally removes OGG1 enzyme copies from the catalytic cycle. It has been suggested that the excised base 8‐oxoG can to some extent initiate the cleavage of the Schiff base by β‐elimination.^3^ (C) TH12117 was developed as a small molecule activator that acts as an organocatalyst. The molecule can replace the excised base and interact with the same amino acid residues.^6^ This interaction enables the molecule to abstract a 2′‐proton more efficiently than 8‐oxoG, initiating a β‐elimination event. The generated reaction product releases the Lys249 residue and OGG1 can enter a new catalytic cycle. In the meantime, the product is directed towards APE1‐ependent repair. This pathway has developed during evolution and deals with lesions arising from 8‐oxoG excision in higher organisms. (D) More potent activator TH10785 has a stronger interaction with the Phe319 and Gly42 residues due to an extended ring system. This enables a more pronounced β‐elimination and in addition a novel δ‐elimination.^6^ Thus, OGG1 can repetitively enter new repair cycles. Although base excision repair initiation is further increased with TH10785, the repair of the generated product is no longer APE1 but rather PNKP1 dependent. This enables a rational overload of a new repair pathway with lesions derived from both 8‐oxoG and AP sites. Light blue – amino acid residues of OGG1; green box – imino group; magenta – aromatic interaction (π‐stacking); purple – H‐bond; Cy – cyclohexyl

After base excision, bifunctional DNA glycosylases commonly generate an enzymatic intermediate that is called Schiff base (Figure 1A,B).^3^ In organocatalysis, activating this iminium species has been one of the most fruitful strategies to enable new and more efficient transformations.^4^ The enzyme 8‐oxoguanine DNA glycosylase 1 (OGG1), for which the cleavage of this intermediate is the rate determining step in the initiation of base excision repair (BER), would be the prime example for activators targeting the enzymatic pocket of a DNA repair protein. This is also due to extended empty space that is formed after the 8‐oxoguanine (8‐oxoG) substrate has been excised.

## 2 INSTALLING A NEW FUNCTION OR INCREASING AN OLD ONE

The creation of activators of OGG1 required knowledge of the enzyme's biochemistry and structural biology, as well as a fine‐tuning of a catalytic nitrogen base as a new variable in medicinal chemistry. Before, we have developed OGG1 inhibitors^5^ and thus we built on this expertise to rationalize small structural changes to lead molecules that enable acid–base chemistry around physiological pH. These synthetic efforts enabled the biochemical and structural basis for OGG1‐targeted organocatalysis.^6^ This included the role as competitive binder to OGG1, however, only in high concentrations and for DNA bearing 8‐oxoG. More importantly, the small molecules enabled OGG1 to perform the cleavage of the intermediate imino species with a 10‐fold increased reaction velocity. When we investigated the enzymatic reaction substrates and products in vitro, we discovered a preference of abasic sites over 8‐oxoG and the generation of an uncommon second product. This new product, 3′‐phosphate modified DNA, corresponded to the generation of a new enzymatic function, a β,δ‐elimination (Figure 1A). Interestingly, a further modulation of the physicochemical properties yielded small molecule activators that only enhanced the formation of the 3′‐phospho unsaturated aldehyde, effectively improving the intrinsic β‐elimination (Figure 1A). The discovery of these different classes of small molecule activators with minor structural changes equals the existence of molecular switches for enzymatic function and allows for full control over which pathway OGG1 funnels its reaction products, as shown in Figure 1A–D.

## 3 OGG1 ACTIVATION AS A STRATEGY TO REROUTE DNA DAMAGE

The two types of OGG1 activator molecules stimulated either the intrinsic OGG1 β‐lyase activity, or the OGG1 β, δ‐lyase activity. Although both classes enhanced OGG1 turnover on 8‐oxoG and abasic sites in vitro, in cells this OGG1 activation increased the kinetics of recruitment and repair of oxidized chromatin. However, depending on the type of activity that OGG1 uses to incise the abasic site upon activation, the BER downstream direction and enzymes required for continuation and completion of the repair process change. Although this moment represents a bottleneck in the general repair process, it is also an opportunity to design specific strategies in which we can take advantage of either type of molecule.

Using activator molecules that stimulate the β‐lyase activity will not alter the repair pathway excessively, as it will continue to use the original downstream enzymes to complete repair. Moreover, although APE1 receives a greater amount of reaction intermediates as a result of OGG1 β‐lyase stimulation, the enzyme is extremely efficient and abundantly expressed in the cell, which ensures a constant supply of intermediates compatible with the terminal enzymes of BER. Thus, we can imagine a scenario in which OGG1 β‐lyase activators reduce levels of oxidative damage without other downstream implications. These types of molecules would be good candidates to develop drugs with a clinical value for the treatment of oxidative stress–related diseases.^7^, ^8^

On the other hand, molecules that stimulate the β,δ‐lyase activity generate a product that can no longer be processed by APE1 and will require additional biochemical modifications to complete BER. Indeed our in vitro studies demonstrated that the enzyme PNKP1 was essential to process the 3′‐phosphate upon installed β,δ‐lyase activity of OGG1. Further, in the absence of PNKP1, BER progression was interrupted. In turn, the generated lesion was not repaired, leading to the formation of DNA strand breaks. In cells, this artificial dependence of PNKP1 can be exploited using specific inhibitors. Thus, by simultaneously treating cells with TH10785 and PNKP1 inhibitors, the repair process is driven to an endless road where 3′‐phosphate intermediates accumulate in the form of unrepairable DNA strand breaks, which are toxic for the cell. This artificial PNKP1 dependency using activators of OGG1 β,δ‐lyase function connects BER to other DNA repair pathways and could thus be exploited as a strategy to treat cancers with high levels of oxidative stress, or bearing inactivating mutations in other DNA repair enzymes.^9^, ^10^

## 4 FUTURE OUTLOOK

The findings outlined in this commentary lay the basis for a new class of biologically active molecules. Increasing enzymatic function in acute disease scenarios or due to occurring mutations is an attractive avenue for the future development of drug candidates. As enzyme‐targeted organocatalysts increase the biochemical reaction rate by a multitude, a limited number of enhanced enzyme copies suffice to, for example, double substrate turnover. This rerouting can either rescue disease burden or overload repair pathways. The strategy stands in sharp contrast to classical inhibition approaches, where a roadblock should almost be complete. Although first of its kind and promising as a technology, additional compound development and applicability in disease settings will be necessary to mature organocatalysts of OGG1 to clinical stages.

## CONFLICT OF INTEREST

TH is listed as inventor on US patent no. WO2019166639A1, which covers OGG1 inhibitors. The patent is fully owned by a non‐profit public foundation, the Thomas Helleday Foundation for Medical Research and TH is a member of the foundation board. TH is a shareholder of Oxcia AB, a company that holds a license to WO2019166639A1. All remaining authors declare that they have no competing interests.
